# Cholera Infantum

**Published:** 1899-01

**Authors:** Grosvenor R. Trowbridge

**Affiliations:** Buffalo, N. Y.


### CHOLERA INFANTUM.

By GROSVENOR R. TROWBRIDGE, A. M., M. D., Buffalo, N. Y.

CHOLERA infantum proper should not be confounded with the serous diarrheas, fermental and those from other causes, but as a disease peculiar to childhood, especially infancy and one due in all probability to a specific microörganism. It is especially likely to occur during the first two or three years of a child's life and during the warm weather. It is, I believe, in a great measure a preventable disease, for among the wealthy classes it is practically unknown.

It occurs especially among the children of the poorer classes of society, who are made liable to it either through neglect or inability on the part of the parents to care for them, and who do not recognise the disease as serious until it is too late.

This disorder in the child is very similar to cholera nostras in the adult, but owing to the lack of resisting power is much more fatal. Cholera infantum is found, as I have stated, especially among the children of the poorer classes, and is due, in the majority of cases, to the following causes: (1) Insufficient and improper food; (2) overcrowding and filthiness; (3) bad or insufficient drainage; (4) long-tubed nursing bottles; (5) teething; (6) excessive heat.

These causes probably do not include the origin of all cases, for cholera infantum will occur when all the above conditions can be eliminated, but for the very large majority these causes will suffice.

In my experience with this disease I wish it understood that every case has occurred in the ninth, eleventh or fourteenth wards of Buffalo, a district inhabited especially by the Polish people, whose ideas on the matter of preventive measures in disease are rather crude and limited. Diarrhea and vomiting in any form whatever are one and the same to them and it is often the physician is blamed, because he is unable to effect a cure in an infant who is in collapse from cholera infantum. The careless feeding of children among the poorer classes is, I believe, the prime cause of cholera infantum and other alimentary disturbances. A woman who is obliged to aid her husband in the support of a large family is unable to properly care for her infant, and the result is it is left to the tender mercies of older children or a neighbor for the entire day, and lack of interest and attention together with the crude ideas of proper food for the baby usually result in gastric and enteric disorders. Even if the mother is at home and has abundance of time to care for her child, ignorance on her part in regard to proper food is often as fatal in its results as if the infant was cared for by others. In a large number of cases I have learned in answer to my invariable inquiry as to what the child receives as nourishment, that it eats everything and this usually includes cabbage, sausage, meat, fish, bread, vegetables of all kinds, fruit, beer, pop, etc., etc. Another extremely common fault to be found with mothers is the habit of feeding a child to keep it quiet. This often results in the overloading of an already too full stomach and a consequent disturbance of the digestive processes. In the majority of cases a little warm (pure) water will suffice to restore the child's good nature, and moreover in many cases will materially aid digestion.

In regard to the second cause, viz., overcrowding and filthiness. I do not consider this as important as that of feeding, yet a weighty reason in many cases. In the district I have mentioned the houses are closely built together, are erected on filled land, which fifteen years ago was for the most part swamp, and even now is improperly drained; are in the majority of cases built without cellars, and families are crowded into them without regard to air space or other sanitary conditions, it frequently being the case that a family of eight or ten occupies two or three small rooms, which serve as sleeping, eating and living apartments. As a rule, there are no running water closets in the houses and the only provision for the disposition of sewage and refuse is the cesspool and privy. I do not wish it thought that I am criticising the Polish inhabitants of Buffalo for the lack of sanitation, for it is not in their power to entirely correct it, but there is no reason why the sewers and openings into them from a number of east side streets should not be kept open and the surface water allowed to drain off, instead of standing for hours and even days as it frequently does. The city of Buffalo is not situated entirely west of Main street. This is a digression, but I believe thoroughly that cholera infantum is often caused by lack of sanitation, and I have in every case endeavored to impress upon the minds of the individual the great importance of cleanliness in every way.

Long-tubed nursing bottles in every instance are a constant menace to the life of an infant, and in every case of affection of any description in the alimentary canal, I have inquired as to whether it was used, and forbidden it if such were the case. It is useless for me to speak of this form of bottle as a cause for cholera infantum, but I wish simply to state that in my practice I know it has been the sole cause for serious intestinal disturbances and consequent death in a number of cases.

Teething is often a factor in alimentary troubles, but I am not inclined to give it as important a place in the causation of cholera infantum as do many writers.

Excessive heat is undoubtedly an important aid in causing intestinal and gastric disturbances, but I do not think it can always be called to account, for it has often been my experience to see cases of cholera infantum exist in extremely cold weather. However, as an adjunct in the causes it is an important factor, for it certainly is true that the vast majority of cases occur during the hot summer months.

*Pathology.*—It has been a source of great regret to me that I have been unable to obtain autopsies in the fatal cases I have seen, for only in two instances have I been able to hold post mortems. In neither of these cases were any gross lesions discoverable, and I believe as Rotch states in his work on Pedriatrics, that "the pathology of cholera infantum has not yet been satisfactorily determined, but it seems to be a noninflammatory disturbance of the whole gastro-enteric tract without any gross lesion beyond a desquamative catarrh, and sometimes hyperemia of the mucous membrane." Dr. Pepper describes the condition as follows: "It is an intense form of acute dyspeptic diarrhea; for few or no lesions of the intestines are found post mortem, and never any of an ulcerative nature. The gravity of the affection seems to be due rather to the associated paralysis of the vaso-motor nerves of the intestine with a resulting profuse discharge of serum, than to any violent enteritis, even of a catarrhal form."

*Symptoms.*—First, a period of restlessness, then abdominal pain, followed by vomiting and this by a profuse diarrhea. The vomitus and stools at first consist of undigested food and afterward principally of serum, in which may be found epithelium and bacteria. The stools and vomitus are at first very offensive, but after the intestinal tract has been emptied of food they are odorless. The abdomen may be slightly distended at first, but later becomes collapsed and soft. The feet and hands become cold, the temperature rises to 103° to 105° F., there is rapid and extreme emaciation, pulse rapid; the urine suppressed; a comatose state or convulsions may follow and death ensue in from twelve to forty-eight hours.

*Prognosis.*—Depends on surroundings, ability and means to care for the child. Always, however, it should be extremely guarded as it often happens that the most extreme cases will recover, while others which are apparently favorable will be intractable to all care and treatment.

*Treatment.*—Is to maintain the strength of the child until the poison causing the disturbance is removed or counteracted. The surroundings of the patient must be taken into consideration and if unsanitary and unfavorable, the child if possible should be removed to the country or at least away from its unsanitary home. It is right here that the great difficulties arise in treating the children of the poorer classes afflicted with such disorders. Usually it means leaving from two to ten other children at home without any care whatever, and so in the majority of these cases the physician is obliged to treat the case as best he can without skilled assistance and sanitary surroundings.

Stimulation at the outset in these cases, notwithstanding that the child is apparently strong, is, I believe, always advisable. This is best obtained by the use of Cognac brandy in small and often repeated doses. As the poison in the alimentary canal is, as a rule, due to the food taken by the child, it is of vast importance that this should be attended to at once. If the child is not in the advanced stage and the excessive weakness is not present a full dose of castor oil, supplemented by high irrigation of the bowel, will often abort an attack.

When the disease is well advanced and the strength of the patient is diminishing rapidly there is considerable risk in the employment of oil or injection and we are obliged to rely on stimulation, proper food and remedial agents which will disinfect the alimentary canal. In regard to food I believe it better in all cases to feed the child artificially and not allow it to nurse. Small quantities of rice or barley water with absolute rest will often counteract the nausea, while warm sterilised water will in a great measure replace the excessive drain caused by the serous stools. Should the temperature reach 104° F. and the surface temperature be raised, sponging frequently with cold water is advisable, but on the other hand should the surface be cold and the temperature per rectum still continue high, warm applications to the body and extremities and enemas of cool water should be employed.

In regard to medication authors do not differ to any great extent. Dr. H. A. Hare in his System of Practical Therapeutics recommends the following, and I have employed them to a considerable extent in my cases:

℞ Acid salicyl.
Sodii biboratis . . . . . . . . . . . . . . . āā g xv.
Aquæ . . . . . . . . . . . . . . . . . . . . . . . mxl.
M et adde.
Tr. aurant cort. . . . . . . . . . . . . . . . . . mv.
Glycerine . . . . . . . . . . . . . . . . . . . . mxv.

℞ Bis-subnit . . . . . . . . . . . . . . . . . . gr. vi.
Tr. opii. deod . . . . . . . . . . . . . . . . . . m i.
Syrupi . . . . . . . . . . . . . . . . . . . . . m xx.
Misturæ cretæ . . . . . . . . . . . . . . . . . ʒ i.

℞ Creasote . . . . . . . . . . . . . . . . . . . . . m ⅛.
Bis-subnit . . . . . . . . . . . . . . . . . . . gr. iij.
Mucilag. acaciæ . . . . . . . . . . . . . . . . m xx.
Aq. cinnamon . . . . . . . . . . . . q. s. f. ℥ i.

Of the first drachm doses may be given and of the two latter one part of the first to three parts of the second and the mixture given in drachm doses.

A favorite prescription, and one which I have used with the best results, is as follows for a child from ten to eighteen months old, the quantities to be increased proportionately:

℞ Salol . . . . . . . . . . . . . . . . . . . . . gr. iii.
Pepsin (pure) . . . . . . . . . . . . . . . . . gr. iiss.
Pancreatin (pure) . . . . . . . . . . . . . . . gr. iiss.
Bis subnit . . . . . . . . . . . . . . . . . . . gr. iii.
Ext. nucis vom . . . . . . . . . . . . . . . . gr. ½.
Sodii bicarb . . . . . . . . . . . . . . . . . . gr. vi.
Ginger powd . . . . . . . . . . . . . . . . . . gr. iss.
M. et div. in chart. No. xii.
Sig.—One every two hours.

Alternating with this about ʒss. of mist. cretæ.

During a period of fifteen months ending October 1, 1897, I treated 321 cases of cholera infantum. They comprised 180 males and 141 females; the average age of males was fifteen months, of the females fourteen months. These were all typical cases of the disease and all occurred among the poorer classes. Twenty cases were comatose when I saw them. The number of deaths was twenty-nine. The treatment was as follows: In 227 cases the powders of salol, pepsin, pancreatin and the like, in conjunction with the chalk mixture were given, with a result of 17 deaths and 210 recoveries; 69 cases were treated with Dr. Hare's formula of creasote, bismuth, etc., with 7 deaths and 62 recoveries, and 25 were given Dr. Hare's second formula (previously mentioned) with the result of 5 deaths and 20 recoveries. The diet and other necessary measures in the way of stimulation, baths and the like were employed in all cases.

I do not hope to advance anything especially new in the treatment of cholera infantum, but having been either fortunate or unfortunate in seeing this considerable number of cases I believe the following points to be of great importance in the care of them: (1) If the child is not too weak, a thorough cleansing of the bowel by means of a laxative and flushing (2) stimulation in every case; (3) carefully regulated feeding; (4) medication should contain an antiseptic to counteract the poison of the disease.

416 ELLICOTT SQUARE.

